# MuSK-Myasthenia Gravis Unmasked by Hydroxychloroquine

**DOI:** 10.1155/2022/4802538

**Published:** 2022-07-15

**Authors:** Shalini Bhaskar, Mohammed Fauzi Bin Abdul Rani

**Affiliations:** Department of Medicine, Universiti Teknologi MARA, Thomson Hospital Kota Damansara, Shah Alam, Malaysia

## Abstract

**Introduction:**

Muscle-specific tyrosine kinase (MuSK) antibody positive myasthenia gravis (MuSK-MG) is a rare clinical disorder, and diagnosing it can be challenging. Most of the patients present with predominant facial, oculo-bulbar, and neck muscle weakness along with respiratory muscle involvement. Such a presentation can be mistaken for bulbar onset motor neuron disease or as one of the rare oculopharyngeal myopathies. *Case Report*. We present a young female patient, who reported to us with neck muscle weakness, ocular and bulbar muscle paralysis, and breathing difficulty. She had been healthy till she was prescribed hydroxychloroquine (HCQ) tablets (400 mg per day) for a malar rash. By the end of the second week after commencing the HCQ therapy, she developed the muscle weakness. Her symptoms began to regress after stopping HCQ and starting steroids, pyridostigmine, and, subsequently, azathioprine. She was negative for anticholinesterase receptor antibodies (AChR-Ab) but was positive for MuSK antibodies (MuSK-Ab).

**Conclusion:**

This report proves that MuSK-MG can also be unmasked by HCQ administration. Awareness of drug-induced/-unmasked MG is important, as failure to do so may result in a severe morbidity and a fatal outcome. The offending drug has to be promptly discontinued, and appropriate treatment should be instituted.

## 1. Introduction

Muscle-specific tyrosine kinase myasthenia gravis (MuSK-MG) or seronegative MG is a distinct subtype of MG affecting 5–8% of all MG patients. MuSK antibodies belong to the IgG4 class of immunoglobulins, which act by direct inhibition of protein function and AChR clustering [1]. Hydroxychloroquine is an immunomodulatory agent which is employed by dermatologists for treating lupus erythematosis. This paper presents the case report of a patient who had been prescribed HCQ elsewhere for a malar rash. She developed myasthenic symptoms which was confirmed to be of the MuSK-Mg type, while being on the drug for two weeks. It is well known that HCQ can potentially worsen or unmask MG in a susceptible individual. Prompt identification of the undesirable side effect of HCQ and institution of appropriate treatment relieved her of her myasthenic symptoms.

## 2. Case Report

A 34-year-old female university lecturer was admitted for bilateral asymmetrical drooping of eyelids with double vision of 4 weeks duration. She also had fluctuating neck muscle weakness which she thought was due to the long hours of online teaching. However, a few days later, her symptoms became progressively worse. Furthermore, she developed dysphonia, dysphagia, and generalized muscle weakness with shortness of breath on exertion.

On enquiry, she admitted to the fact that she had recently developed maculo-papular rash over her cheeks which was diagnosed as photosensitivity rash. The attending dermatologist prescribed her hydroxychloroquine (HCQ) 400 mg per day orally which she took for two weeks prior to the emergence of the neurological symptoms. She had no significant past medical illnesses except for 2 episodes of acute simple cystitis that resolved with a single dose of oral fosfomycin, and on both the occasions, no myasthenic symptoms were precipitated. Fosfomycin is a phosphonic acid derivative and has not been listed as an agent that aggravates or triggers myasthenic symptoms in a susceptible individual.

**Figure 1:**
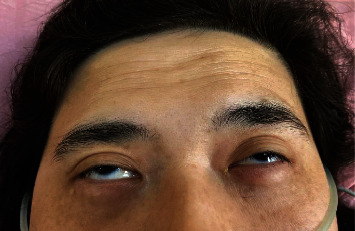
Bilateral partial ptosis with overaction of the frontalis muscle.

On examination, she was tachypneic with a respiratory rate of 28 breaths/min. Her oxygen saturation was fluctuating between 86% and 92% necessitating nasal oxygen administration. She had neck muscle weakness and asymmetrical external ophthalmoplegia (Figure 1). Swallowing function was impaired resulting in choking for fluids. There was fluctuating muscle fatigue and double vision on various eye gaze positions. The proximal limb muscle power over both upper and lower limbs was 3/5, and she was unable to sit upright for long. The deep tendon reflexes and sensory examination were normal.

**Figure 2:**
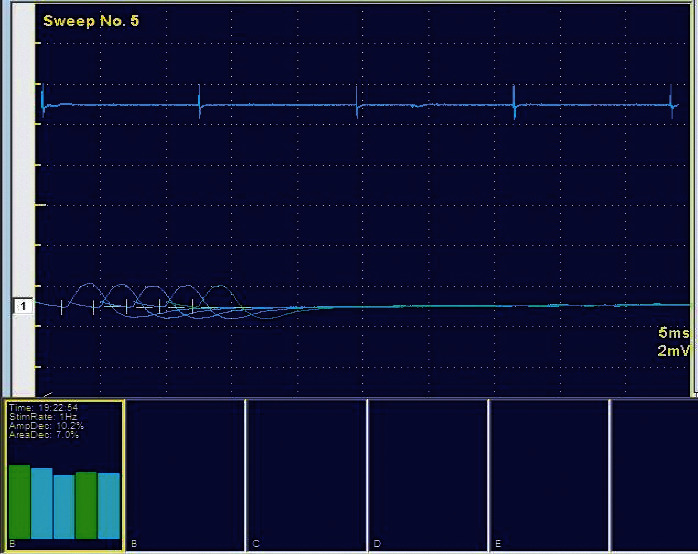
Decremental response of 10% seen on repetitive stimulation of the facial nerve.

Based on the history and clinical signs and symptoms, a diagnosis of myasthenia gravis was made. This was substantiated by the repetitive nerve stimulation test, wherein at 1 Hz rate stimulation, the orbicularis oculi muscles showed a decremental response consistent with the diagnosis of myasthenia gravis (Figure 2). The AChR antibody titer was reported to be normal, whereas the MuSK antibody titer was high. Computed tomography (CT) of her thorax did not show any thymic enlargement. Blood tests for connective tissue disorders such as the antinuclear antibody (ANA), double-stranded DNA (dsDNA), and extractable nuclear antigen (ENA) were negative.

Based on the suspicion of HCQ-triggered myasthenia, the patient was asked to stop the HCQ tablet. She was treated concomitantly with intravenous methylprednisolone (1 gram) daily for 5 days followed by oral prednisolone (30 mg) daily along with pyridostigmine tablet (60 mg) thrice a day. After 10 days, the prednisolone dose was gradually tapered off, and oral azathioprine (50 mg daily) was started. This clinical presentation prompted us to arrive at a final diagnosis of MuSK- MG unmasked by HCQ treatment.

## 3. Discussion

Myasthenia gravis (MG) is the most common type of neuromuscular transmission disease, and 85% of the patients have autoantibodies against acetylcholine receptors (AChRs). Muscle-specific receptor tyrosine kinase myasthenia gravis (MuSK-MG) is reported in about 5–8% of MG patients and MuSK.

Antibodies are detected in approximately 37% of generalized AChR antibody-negative myasthenia gravis [2].

This disease is predominantly seen in females in their 3^rd^ decade with prominent facial and bulbar involvement and more frequent crises. The onset is usually acute with rapid progression within a few weeks. Bulbar muscle involvement has been demonstrated in up to 80% of the MuSK-MG patients, resulting in dysarthria, dysphonia with nasal voice, dysphagia, and masticatory difficulty. Severe neck muscle weakness presenting as head drop is a feature in Musk-MG. Thus, the patient may be easily misdiagnosed as having bulbar onset amyotrophic lateral sclerosis, oculopharyngeal muscular dystrophy, or mitochondrial myopathy.

Case reports on chloroquine-induced MG have appeared in the literature ever since 1981, and most patients have had symptomatic recovery and the disappearance of the AChR antibody after withdrawing chloroquine [3, 4]. Varan et al. in 2015 reported a patient diagnosed with systemic lupus erythematosus developing myasthenia gravis due to hydroxychloroquine [5]. In the era of the COVID-19 pandemic, when HCQ has been widely used as prophylactic treatment, Elavarsi and Goyal [6] reported the exacerbation of myasthenic symptoms in a young AChR-positive myasthenic lady treated with HCQ and emphasized the need for awareness of the possibility of HCQ-induced MG. In 2012, Jallouli et al. [7] published a case series of 17 patients with MG and SLE with a special focus regarding hydroxychloroquine usage. It was found that in 8 patients, myasthenia occurred after the initiation of HCQ for the treatment of SLE. However, only one of them was presumed to be due to HCQ, and this patient had a rapid development of myasthenic symptoms though not associated with antibodies against AChR. There was resolution of symptoms following withdrawal of the drug; however, rechallenge with HCQ was not done. Chloroquine, a precursor of HCQ, has been associated with the emergence of myasthenia through production of AChR antibodies causing immune checkpoint inhibitors (ICI) leading to exacerbation of MG through direct effect on neuromuscular transmission [8].

HCQ has been used for several decades for various connective tissue diseases, and it has gained popularity over the last one year as a drug for the prophylaxis of COVID-19 infection. These classes of drugs prevent the biocrystallization of heme and impaired neuromuscular transmission both at the presynaptic and postsynaptic levels [9]. A systematic review by Sheikh et al. [10] summarizes the various drugs which can cause de novoMG, MG exacerbation, or MG-like symptoms in nonmyasthenic patients.

Whether HCQ can trigger MuSK-MG is also not clearly known, and no reports on such cases have been forthcoming in the literature. Therefore, our case is a rare presentation of MuSK-MG being unmasked by HCQ.

## 4. Conclusion

Our case is rather unique, and it proves to show that HCQ can unmask MuSK-MG also, which has not been hitherto reported in the literature. From a clinical perspective, it is of utmost importance for the treating physician to be aware of such adverse effects of certain drugs such as HCQ and their consequences on the neuromuscular junction. The possibility of MuSK-MG should be entertained in AChR-Ab-negative MG patients, and antibody titer estimation for the MuSK antibody should be performed.

## Data Availability

The data that support the findings of this study are available on request from the corresponding author.
